# Awake versus intubated video-assisted thoracoscopic surgery for pleural disease: a retrospective cohort study from a single tertiary center

**DOI:** 10.3389/fsurg.2025.1635663

**Published:** 2025-07-17

**Authors:** Omer Faruk Saglam, Burcu Kilic, Merve Ekinci Fidan, Nevzat Cem Sayilgan, H. Volkan Kara, Akif Turna, Kamil Kaynak, Ezel Ersen

**Affiliations:** ^1^ Department of Thoracic Surgery, Istanbul University-Cerrahpaşa, Cerrahpaşa Faculty of Medicine, Istanbul, Türkiye; ^2^ Department of Thoracic Surgery, Yedikule Chest Diseases and Thoracic Surgery Training and Research Hospital, Istanbul, Türkiye; ^3^ Department of Anesthesiology and Reanimation, Istanbul University-Cerrahpaşa, Cerrahpaşa Faculty of Medicine, Istanbul, Türkiye

**Keywords:** awake video-assisted thoracic surgery, minimally invasive thoracic surgery pleural disease, pleural effusion, spontaneous respiration, pleural disease

## Abstract

**Introduction:**

Awake video-assisted thoracoscopic surgery (A-VATS) has gained increasing attention as an alternative to classical intubated VATS (I-VATS), particularly in patients with comorbidities that have increased the risk of surgery under general anesthesia. This study aimed to compare the perioperative and postoperative outcomes of patients who underwent A-VATS vs. I-VATS for pleural diseases.

**Methods:**

This is a retrospective cohort study including patients who underwent A-VATS (*n* = 22) and I-VATS (*n* = 37) for pleural diseases between July 2015 and March 2023 at a single tertiary step medical center. Patients considered unsuitable or at high risk for I-VATS due to anesthetic risk or comorbidities were allocated to the A-VATS group. Demographic characteristics, comorbidities, risk scores, spirometry results, surgical outcomes, anesthesia satisfaction, surgical and other complications, and laboratory parameters were analyzed.

**Results:**

A-VATS had significantly lower NRS scores at all postoperative timepoints (1, 12, 48 h; *p* < 0.01) and reduced NSAID use (*p* = 0.04), whereas opioid use was similar between the groups. The incidence of postoperative atelectasis was higher in the I-VATS group (*p* < 0.001). Earlier oral intake, mobilization, and return of bowel function were observed in the A-VATS group (all values compared were *p* < 0.001). Although the hospital stay was longer in the A-VATS group (5.0 vs. 2.0 days; *p* = 0.01), there was no difference in hospitalization costs between the groups (*p* > 0.05). There was no difference in the overall complication rates (*p* > 0.05). Hematological and biochemical parameter changes were similar between the groups.

**Conclusions:**

A-VATS is a potential feasible alternative in appropriate patients who have a higher risk with I-VATS. A-VATS offers favorable outcomes in terms of postoperative pain control and better recovery so may replace I-VATS. However, its use requires careful patient selection and perioperative planning due to the occurrence of severe complications in some cases. Prospective randomized, patient matched larger and multiple study groups are needed and in our future plan to confirm these findings and optimize the perioperative and postoperative protocols for A-VATS.

## 1 Introduction

Video-assisted thoracic surgery (VATS) is performed through one or more port sites using a videothoracoscope without rib spreading using a retractor or cutting (1). General anesthesia carries a significant risk of ventilator dependence, along with increased complications, morbidity, and mortality in patients with severe respiratory diseases (2–4). Several studies have indicated that general anesthesia for classically intubated VATS (I-VATS) may hinder clinical recovery in patients because of a potentially higher incidence of respiratory complications linked to postoperative residual neuromuscular blockade (2, 3). Awake VATS (A-VATS) may reduce the side effects associated with intubation, general anesthesia, and single-lung ventilation (5).

By avoiding excessive use of muscle relaxants and mechanical ventilation, A-VATS may prevent a range of complications including lung and diaphragmatic dysfunction with related tissue injuries, sore throat, mucosal ulceration, and bronchospasm due to mechanical irritation (2, 6). It may also mitigate the adverse effects associated with residual muscle blockade that can lead to temporary upper airway weakness, airway obstruction, and hypoxemia (2, 3). Additionally, A-VATS is associated with a lower prevalence of latent aspiration, which can lead to pneumonia (7, 8). The elimination of residual neuromuscular effects and alleviation of inhibitory reflexes on the diaphragm may enhance mucociliary clearance, thereby improving pulmonary function (9). Absolute contraindications include patient refusal to consent to the procedure and clinical assessment by the surgical team, deeming the patient unsuitable for this technique (10).

In this study, we aimed to evaluate the impact of A-VATS on perioperative and postoperative parameters in the patient group. We retrospectively analyzed the data of patients who underwent A-VATS and I-VATS for pleural and pericardial clinical occasions that need diagnostic or therapeutic surgical intervention. We sought to compare and clarify potential advantages of the A-VATS procedure in the general patient population, in whom general anesthesia is typically not possible or highly risky.

## 2 Materials and methods

### 2.1 Study design and patient selection

This study was designed as a retrospective cohort analysis. This study was approved by the Institutional Clinical Research Ethics Committee of our university (Date and Number: 22/03/2023-649839). The research group consisted of patients who underwent surgical intervention for pleural indications at the thoracic surgery department of a tertiary academic medical center between July 2015 and March 2023. Patients in the study group underwent awake video-assisted thoracoscopic surgery (A-VATS). The control group included patients who underwent intubated VATS (I-VATS) for similar pleural indications. Patients with inaccessible data were excluded from the study. Ultimately, 22 patients who underwent A-VATS for pleural indications were included in the study group, and 37 patients who underwent I-VATS for comparable indications constituted the control group.

Patients were required to have up dated thoracic computed tomography (CT) scans, complete blood count (CBC) and serum biochemical parameters. Potential risks and preoperative comorbidities were also recorded. Patients were followed up preoperatively, intraoperatively, and postoperatively in accordance with the Enhanced Recovery After Surgery (ERAS) protocol (11).

This study was conducted in accordance with the principles of the Declaration of Helsinki. Patient confidentiality was maintained throughout the study, and no identifiable data were used in the analysis or publication. None of the researchers declare any potential conflict of interests related to this study.

### 2.2 Indications and procedures

The patients were evaluated based on clinical symptoms, imaging findings, and laboratory data. The most common indication for surgery was pleural effusion, followed by pleural thickening, loculated effusion, fibrothorax, and pericardial window opening. The procedures were individualized according to pathology and included pleural drainage, pleural and mass biopsies, deloculation, partial decortication or pleurectomy, debridement, and lymph node sampling. Pericardial biopsy and window formation were performed in selected cases to address the suspected malignant or inflammatory processes. Patients who presented with coexisting conditions, such as pleural thickening with fibrothorax, often required more extensive and additional interventions (e.g., decortication or pleurectomy), and multiple procedures were performed concurrently and tailored to the underlying pathology.

The decision to perform A-VATS or I-VATS to a patient was made by an experienced multidisciplinary team, including thoracic surgeons and anesthesiologists. Each patient's functional status, comorbidities, and anesthetic risk in total effected on the choice. A-VATS was selected for patients in whom general anesthesia or intubation was not feasible or posed a high risk due to compromised respiratory function or poor general condition. These decisions were further supported by elevated scores on the American Society of Anesthesiologists (ASA) physical status classification and the Charlson Comorbidity Index (12). In accordance with the ongoing debate in the literature regarding “absolute contraindications”, no specific contraindications were defined in this study, except for the presence of vital sign abnormalities resulting in hemodynamic instability or the lack of informed consent (10).

### 2.3 Anesthesia and operating room protocol

All patients and family members were preoperatively informed about the anesthesia method and the possibility of conversion to general anesthesia if needed with written informed consent. Standard monitoring included electrocardiography, peripheral oxygen saturation (SpO₂), and invasive arterial pressure via radial artery cannulation during the procedure.

Sedation was achieved using intravenous midazolam (0.03 mg/kg) or dexmedetomidine (1 µg/kg over 15 min). Supplemental oxygen was administered via a nasal cannula or face mask to maintain SpO₂ above 90%. In cases that required deeper sedation or inadequate analgesia, additional titrated doses of remifentanil, fentanyl, ketamine, or propofol were administered.

Regional anesthesia was performed using either a paravertebral block or erector spinae plane block, depending on the patient's anatomical considerations, comorbidities, and surgical site. Local anesthetic mixtures typically include bupivacaine, fentanyl, morphine, and adjuvants such as dexamethasone or epinephrine. These were administered as either a single-shot block or continuous catheter infusion. Regional anesthesia catheters were removed the day after surgery, as the final analgesic dose was applied.

The patients were positioned according to the procedural requirements in the lateral decubitus, semi-lateral, or semi-Fowler positions. All airway and thoracoscopic equipment, including videolaryngoscopes and endotracheal tubes, were prepared in advance to allow for rapid conversion to general anesthesia, if necessary.

Surgical approaches (uniportal, biportal, triportal) were selected based on individual surgeon experience, patient anatomy, and specific clinical indications. A 10 mm 30° thoracoscope and standard thoracoscopic instruments were used. At the end of the procedure, a single chest tube was inserted through the lowest port site and connected to an underwater drainage system. Chest tubes were removed if there was no air leak and the drainage volume was less than 200 ml in the 24 h after the second postoperative day.

### 2.4 Data collection, outcome measures and endpoints

Demographic and clinical variables; preoperative, perioperative, and postoperative laboratory, monitoring, and surgical outcome data; CBC and serum biochemistry; and cost data were collected and analyzed. Preoperative laboratory values were obtained 24 h before surgery. Postoperative laboratory data were collected within 1 h of the completion of the procedure. Pulmonary function tests were assessed preoperatively via spirometry, including FEV1, FVC, and the FEV1/FVC ratio, following standard American Thoracic Society/European Respiratory Society protocols (13). ASA, Mallampati (14), and metabolic equivalent of task (MET) (15) scores were determined as part of the pre-anesthesia evaluation. The Charlson Comorbidity Index, Cardiac Risk Index (16), and Pulmonary Risk Index (17) were also recorded. Postoperative pain was evaluated using the Numeric Rating Scale (NRS), scored from 0 (no pain) to 5 (worst imaginable pain) (18). Postoperative complications were recorded as any deviation from normal recovery and classified using the Clavien–Dindo grading system (19). Grade I complications were categorized as minor. Anesthesia satisfaction was assessed using a 5-point Likert-type scale ranging from 1 (very dissatisfied) to 5 (very satisfied) (20). Cost analysis encompassed all direct hospitalization expenses, including surgical and anesthesia fees, medication and analgesia requirements, nursing care, diagnostic tests, consumables, and overall hospital accommodation. Intensive care unit admissions, when required, were also included. Cost data were extracted retrospectively from hospital financial records and presented in Turkish Liras (TRY).

The primary outcomes were postoperative pain scores (NRS), analgesic requirements, complication rates, duration of hospitalization and chest drainage, and early recovery indicators including time to mobilization, oral intake, and return of bowel function. Secondary outcomes included demographic characteristics, preoperative assessments, and changes in laboratory parameters.

### 2.5 Statistical analyses

Statistical analyses were performed using IBM SPSS Statistics for Windows, Version 27 (IBM Corp., Armonk, NY, USA). Descriptive statistics are presented as mean ± standard deviation for normally distributed continuous variables and as median with interquartile range (Q1–Q3) for non-normally distributed data. Categorical variables were summarized using frequencies and percentages. Kolmogorov–Smirnov and Shapiro–Wilk tests were used to assess the normality of continuous variables. Between-group comparisons of continuous variables were performed using the independent samples *t*-test for normally distributed data and the Mann–Whitney *U* test for non-normally distributed data. The paired sample *t*-test or Wilcoxon signed-rank test was used for within-group comparisons of preoperative and postoperative laboratory parameters, depending on the distribution. Categorical variables were compared using the chi-square test or Fisher's exact test, as appropriate, based on the expected cell frequencies. Statistical significance was set at *p* < 0.05. Only univariate analyses were performed. Although propensity score matching is frequently employed in retrospective studies to reduce selection bias and confounding factors, it was not feasible because of the small sample size.

## 3 Results

### 3.1 Demographic characteristics and comorbidities

Demographic characteristics, including age, sex, BMI, and smoking status, were comparable between groups (*p* > 0.05). Chronic renal failure, preoperative sinus tachycardia, and a history of pleural or pulmonary surgery were more frequent in the A-VATS group (*p* < 0.05). A-VATS patients also more commonly had reduced functional capacity (MET ≤4; 68.2% vs. 35.1%; *p* = 0.01). Although a greater proportion were classified as ASA III, ASA score distribution was not significantly different. Charlson Comorbidity, Cardiac and Pulmonary Risk indices, and Mallampati scores were similar (*p* > 0.05). Preoperative FEV1% and FVC% values were lower in the A-VATS group, but the differences were not statistically significant (Table 1).

**Table 1:** Demographic characteristics, comorbidity, risk scales, spirometry values of the patients.

Demographic and clinical parameters	I-VATS (*n*:37)	A-VATS (*n*:22)	*p*
Age (mean ± SD)	55.5 ± 12.5	53.6 ± 20.04	0.65
Male (*n*, %)	21 (56.8%)	14 (63.6%)	0.60
BMI (kg/m^2^) (mean ± SD)	27.2 ± 6.0	24.3 ± 3.9	0.05
Smoking (*n*, %)	24 (64.9%)	15 (68.2%)	0.79
Ex-smoker	15 (40.5%)	11 (50.0%)	0.48
Active	9 (24.3%)	4 (18.2%)	
Cigarettes (pack.year) (median, Q1-Q3)	22.5 (11.3–40.0)	15 (6.0–40.0)	0.56
Comorbidities (n, %)	36 (97.3%)	22 (100.0%)	1.00
Pulmonary	25 (67.6%)	18 (81.8%)	0.23
Cardiac	12 (32.4%)	10 (45.5%)	0.32
Psychiatric/Neurological disease	7 (18.9%)	1 (4.5%)	0.12
Diabetes mellitus	9 (24.3%)	4 (18.2%)	0.58
Chronic kidney failure	0 (0.0%)	4 (18.2%)	**0** **.** **02**
ASA Score (*n*, %)			
I	5 (13.5%)	2 (9.1%)	0.11
II	21 (56.8%)	8 (36.4%)	
III	11 (29.7%)	11 (50.0%)	
IV	0 (0%)	1 (4.5%)	
Mallampati Score (*n*, %)			
I	15 (40.5%)	10 (45.5%)	0.08
II	15 (40.5%)	12 (54.5%)	
III	5 (13.5%)	0 (0.0%)	
IV	2 (5.4%)	0 (0.0%)	
METS (*n*, %)			
<4	13 (35.1%)	15 (68.2%)	**0** **.** **01**
≥4	24 (64.9%)	7 (31.8%)	
Charlson C. risk (median, Q1–Q3)	1.0 (0.0–2.0)	2.0 (0.0–3.3)	0.12
High (*n*. %)	14 (37.8%)	12 (54.5%)	0.21
Low (*n*. %)	23 (62.2%)	10 (45.5%)	
Cardiac risk index (*n*, %)			
0	0 (0.0%)	1 (4.5%)	0.13
1	35 (100.0%)	19 (86.4%)	
2	0 (0.0%)	2 (9.1%)	
Pulmonary risk score (*n*, %)			
0	8 (21.6%)	6 (27.3%)	0.86
1	18 (48.6%)	7 (31.8%)	
2	9 (24.3%)	7 (31.8%)	
3	2 (5.4%)	2 (9.1%)	
Spirometry (median, Q1–Q3)			
FEV1 (liter)	1.77 (1.52–2.16)	1.47 (0.88–2.45)	0.19
FEV1%	67.00 (57.50–77.25)	57.00 (42.00–78.00)	0.11
FVC (liter)	2.38 (2.04–3.04)	1.92 (1.44–3.62)	0.18
FVC % (mean ± SD)	73.67 ± 18.76	64.07 ± 18.76	0.11
FEV1/FVC	77.00 (68.75–82.49)	77.0 (67.02–81.00)	0.81

Bold values represent statistically significant results (*p* < 0.05).

I-VATS, intubated video-assisted thoracoscopic surgery; A-VATS, awake video-assisted thoracoscopic surgery; SD, standard deviation; BMI, body mass index; ASA, American Society of Anaesthesiologists physical status classification; METS, metabolic equivalent of task; FEV1, forced expiratory volume in 1 second; FVC, forced vital capacity.

The indications and surgical procedures are presented in Table 2.

**Table 2:** Indications and procedures.

Surgical indications and procedures	I-VATS (*n*:37)	A-VATS (*n*:22)	*p*
Indications (*n*, %)			
Pleural effusion	30 (81.1%)	20 (90.9%)	0.46
Pleural thickening	27 (73%)	11 (50%)	0.07
Loculation	9 (24.3%)	8 (36.4%)	0.41
Fibrothorax	2 (5.4%)	5 (22.7%)	0.09
Empyema	2 (5.4%)	4 (18.2%)	0.18
Pericardial effusion	0 (0%)	1 (4.5%)	0.37
Procedures (*n*, %)			
Drainage	27 (73%)	18 (81%)	0.44
Pleura biopsy	36 (15%)	15 (68.2%)	**0** **.** **03**
Deloculation	7 (18.9%)	8 (36.4%)	0.14
Pleural debridement	3 (8.1%)	9 (40.9%)	0.06
Pleural mass biopsy	1 (2.7%)	9 (40.9%)	1.00
Partial Decortication/Pleurectomy	1 (2.7%)	4 (18.2%)	0.06
Lymph node biopsy	1 (2.7%)	1 (4.5%)	1.00
Pericardial biopsy	0 (0%)	1 (4.5%)	0.37
Pericardial fenestration	0 (0%)	1 (4.5%)	0.37

Bold values represent statistically significant results (*p* < 0.05).

I-VATS, intubated video-assisted thoracoscopic surgery; A-VATS, awake video-assisted thoracoscopic surgery.

### 3.2 Perioperative outcomes, hemodynamic and vital signs and complications

The surgical outcomes and results are presented in Table 3.

**Table 3:** Post-surgery outcomes.

Postoperative and perioperative parameters	I-VATS (*n*:37)	A-VATS (*n*:22)	*p*
NRS for pain (median, Q1–Q3)			
1st hour	4.0 (3.0–5.0)	2.0 (2.0–3.0)	**0** **.** **002**
12th hour	3.0 (2.0–4.0)	2.0 (1.0–3.0)	**0** **.** **001**
48th hour	2.0 (2.0–3.0)	1.0 (0.0–4.0)	**<0** **.** **001**
Need for NSAID (*n*, %)	32 (86.5%)	14 (63.6%)	**0** **.** **04**
Need for Opioid (*n*, %)	18 (48.6%)	9 (40.9%)	0.56
Local Analgesia (*n*, %)	1 (2.7%)	18 (97.3%)	**<0** **.** **001**
Regional Block (*n*, %)	1 (2.7%)	17 (77.3%)	**<0** **.** **001**
Pre-operative/Per-operative Δ Heart Rate bpm	−3.0 (−14.5–2.0)	3.5 (−10.0–19.0)	**0** **.** **04**
Postoperative Systolic Blood Pressure mmHg (mean ± SD)	127.08 ± 19.75	115.82 ± 21.4	**0** **.** **04**
Postoperative Diastolic Blood Pressure mmHg (mean ± SD)	78.73 ± 12.54	70.5 ± 12.68	**0** **.** **02**
Postoperative Body Temperature °C	35.8 (35.7–36.2)	36.4 (36.0–36.6)	**0** **.** **003**
Pre-operative/Per-operative ΔSpo_2_	2.0 (1.0–3.0)	2.5 (0.0–5.0)	0.97
Complication (*n*, %)	36 (97.3%)	21 (95.5%)	1.00
Clavien-Dindo Class (*n*, %)			
I	29 (78.4%)	15 (68.2%)	0.57
II	7 (18.9%)	2 (9.1%)	
IIIA	1 (2.7%)	2 (9.1%)	
IIIB	0 (0.0%)	1 (4.5%)	
IV	0 (0.0%)	2 (9.1%)	
Atelectasis on Postoperative Day 0 Chest x-ray (*n*, %)	26 (70.3%)	5 (22.7%)	**<0** **.** **001**
Postoperative sore throat (*n*, %)	26 (70.3%)	2 (9.1%)	**<0** **.** **001**
Surgical Outcomes (median, Q1–Q3)			
Anaesthesia Satisfaction Score	3.14 (3.0–3.0)	5.0 (5.0–5.0)	**<0** **.** **001**
Total Duration of Anesthesia (min)	105.0 (90.0–140.0)	92.5 (66.3–125.0)	0.055
Duration of Surgery (min)	60.0 (45.0–85.0)	50.0 (35.0–65.0)	0.06
Length of Stay (days)	2.0 (1.5–4.5)	5.0 (3.0–14.0)	**0** **.** **01**
Duration of Chest Drain (days)	2.0 (1.0–4.0)	4.0 (2.5–8.5)	**0** **.** **01**
Duration of Air Leak (days)	0.0 (0.0–0.0)	0.0 (0.0–1.0)	0.15
Total Drainage (ml)	400.0 (150.0–575.0)	350.0 (225.0–875.0)	0.39
Oral Intake (min)	280.0 (165.0–445.0)	65 (38.8–120.0)	**<0** **.** **001**
Ambulation (min)	300.0 (180.0–505.0)	77.5 (48.8–182.5)	**<0** **.** **001**
Flatulance/Defecation (min)	1,540.0 (1,323.0–2,450.0)	810 (375.0–1,085.0)	**<0** **.** **001**
Cost (Turkish Liras)	4,623 (3,275–5,771)	3,990 (2,264–6,145)	0.39

Bold values represent statistically significant results (*p* < 0.05).

I-VATS, intubated video-assisted thoracoscopic surgery; A-VATS, awake video-assisted thoracoscopic surgery; NRS, numeric rating scale; NSAID, nonsteroidal anti-inflammatory drugs; bpm, beats per minute; SD, standard deviation; Spo2, oxygen saturation.

Postoperative pain scores were significantly lower in the A-VATS group at all time points (1, 12, 48 h; *p* ≤ 0.002). NSAID use was higher in the I-VATS group (*p* = 0.04), while opioid use was similar (*p* = 0.57). Local analgesia and regional blocks were used more frequently in the A-VATS group (both *p* < 0.001) (Table 3).

Postoperatively, the A-VATS group had higher heart rate changes (*p* = 0.04), lower systolic and diastolic blood pressures (both *p* = 0.04), and higher body temperatures (*p* = 0.003). SpO₂ changes showed no significant difference between groups (*p* = 0.96) (Table 3).

Overall complication rates and Clavien–Dindo classification distributions were similar between groups (*p* > 0.05). However, severe complications (grades IIIB–IV) occurred only in the A-VATS group (14.6%), including pneumothorax requiring tube thoracostomy, intraoperative hypotension necessitating conversion to general anesthesia, and postoperative bleeding requiring ICU admission. Postoperative sore throat and microbial culture positivity were more frequent in the I-VATS group. Other symptoms and complications showed no significant differences. Atelectasis (70.3% vs. 22.7%; *p* < 0.001) and sore throat (70.3% vs. 9.1%; *p* < 0.001) were significantly more common in the I-VATS group (Table 3).

Anesthesia satisfaction was significantly higher in the A-VATS group (*p* < 0.001). A-VATS patients also achieved earlier oral intake, mobilization, and return of bowel function (all *p* < 0.001). Drain duration and hospital stay were longer in the A-VATS group (*p* = 0.01), while total drainage volume and air leak duration were similar (*p* > 0.05). Hospitalization costs did not differ significantly between groups (*p* = 0.39) (Table 3).

### 3.3 Hematologic and biochemical parameters

The postoperative inflammatory response, as measured by neutrophil parameters, was significantly greater in the I-VATS group compared to the A-VATS group. The increase in absolute neutrophil count (×10^3^/µl) was higher in the I-VATS group (median: 100.0 [27.9–183.6]) than in the A-VATS group (36.8 [−93.2–238.1]; *p* = 0.04). Similarly, the change in neutrophil percentage was greater in the I-VATS group (19.2% [4.7–28.0]) compared to the A-VATS group (8.8% [1.5–20.1]; *p* = 0.04). The relative increase in neutrophil percentage was also significantly higher in the I-VATS group (28.7% [6.2–44.5]) vs. the A-VATS group (9.2% [−90.1–58.0]; *p* = 0.02). No significant differences were observed between groups in other hematologic or biochemical parameters.

## 4 Discussion

Although the patients selected for A-VATS in our study were deemed unsuitable for general anaesthesia during the preoperative evaluation, A-VATS exhibited more favorable physiological outcomes in both the perioperative and postoperative periods, enabling a quicker and more comfortable return to normal function.

There is no absolute consensus regarding the patient population for which A-VATS should be preferred or avoided. A-VATS is generally chosen for patients in whom the surgical outcome is expected to be satisfactory and for those who are considered technically suitable for the procedure. In one retrospective study analyzing A-VATS cases, it was reported that patients aged 21–104 years and weighing between 40 and 181 kg could tolerate A-VATS under sedation and local anaesthesia. Patients should not be excluded from A-VATS based on age, height, weight, BMI, or comorbidities (10). In our study, there were no differences between the groups in terms of age, sex, height, weight, BMI, ASA and Mallampati scores, smoking status, active smoking, or the presence of any comorbidity. However, the lower MET score observed in the A-VATS group than in the intubated group suggests that A-VATS patients had reduced functional capacity.

Avoidance of general anaesthesia and double-lumen intubation facilitates the preferential use of A-VATS in patients considered unfit for general anaesthesia, underscoring the importance of accounting for variations in the overall condition, comorbidities, and ASA grading (21). Several studies have successfully performed A-VATS in patients with different ASA classes (4, 10, 21, 22). In our study, ASA and Mallampati scores did not differ between the groups, while the only patient with an ASA 4 status underwent awake surgery.

To anticipate potential incompatibility between the patient and the surgical team during the procedure, we compared the prevalence of psychiatric or neurological disorders, which could impact mental and motor cooperation between the groups; no significant difference was found.

In the study by Gökçe et al., which assessed drainage and pleural biopsies in patients with undiagnosed exudative pleural effusion, the mean Charlson Comorbidity Index was higher in the A-VATS group compared to the I-VATS group (5.24 [2.27] vs. 2.3 [2.09], *p* <0.001) (23). Similarly, in a retrospective study by Cai et al. involving patients with lung cancer, comorbidities were noted in 9.3% of the I-VATS group and 7.7% of the A-VATS group, with no intergroup differences (24). In our study, while no differences were observed between the groups regarding the Charlson Comorbidity, Cardiac, and Pulmonary Risk indices, or the presence of respiratory, cardiac, or diabetes mellitus comorbidities, the A-VATS group exhibited a higher prevalence of previous pleural or parenchymal interventions, sinus tachycardia, and chronic renal failure. Although the overall comorbidity burden did not significantly differ between the groups, these findings suggest that patients undergoing A-VATS represent a more vulnerable population.

Studies evaluating A-VATS cases have reported that, within a subgroup of 502 resections in patients with severely compromised pulmonary status, preoperative FEV₁ was less than 0.8 L—and in some instances even below 0.5 L (25–27). For example, a case report described an A-VATS bullectomy performed for a pneumothorax in a female patient with advanced respiratory dysfunction, whose spirometry values were FVC 0.89 L (32.8%), FEV₁ 0.82 L (93.18%), and FEV₁/FVC of 93.12 (28). In our series, although the A-VATS group exhibited lower preoperative spirometry values (minimum FEV₁/FVC of 38.0 vs. 43.0, FEV₁ of 0.4 vs. 0.6 L, FVC of 0.8 vs. 1.0 L, median FEV₁% of 57% vs. 67% with *p* = 0.11, and mean FVC% of 73.67% vs. 64.07% with *p* = 0.11), these differences were not supported by the *p*-values obtained. Nonetheless, the lower spirometry values observed in the A-VATS group may suggest that this group consisted of patients who required more careful evaluation of their respiratory function.

Although some studies have not found a statistically significant difference in the visual analog scale (VAS) scores between the A-VATS and I-VATS groups (29, 30), systematic reviews and meta-analyses have indicated that the VAS scores are significantly lower in the A-VATS group (31, 32). Furthermore, several studies have shown that postoperative analgesic consumption was significantly lower in the A-VATS group than in the I-VATS group (33). Effective pain control achieved with locoregional anaesthesia techniques in A-VATS—without the narcotic complications associated with general anaesthesia—seems to contribute to a notable reduction in postoperative pain (34–36). Postoperative pain was assessed using the Numeric Rating Scale (NRS; 1–5). NRS scores at 3, 12, and 48 h, as well as NSAID requirements, were significantly lower in the A-VATS group, likely reflecting the more frequent use of regional anesthesia and local analgesia, including catheter-based techniques maintained for up to 24 h postoperatively. Opioid use did not differ between groups. Surgical approaches (uniportal, biportal, triportal) were chosen based on surgeon preference and clinical context; however, due to the limited sample size, their impact on pain and recovery could not be analyzed and warrants investigation in future studies.

General anesthesia, through both inhalation and intravenous agents, reduces cardiac contractility, ejection fraction, heart rate, and heart rate variability via parasympathetic activation pathways (37, 38). General anaesthesia may lead to fluctuations and reductions in blood pressure owing to decreased systemic vascular resistance, diminished baroreflex responses, changes in the patient's volume status, and alterations in cardiac preload and output (39). A-VATS, which preserves autonomic regulation and normal cardiovascular responses during surgery and employs a lighter sedation regimen that generally incorporates local anaesthesia, helps mitigate the stress response associated with general anaesthesia and intubation by reducing fluctuations in heart rate and blood pressure (40, 41). In a decortication study, the heart rate in the A-VATS group was reported to be significantly lower (42). Various studies have shown that, due to the avoidance of muscle relaxants and heavy sedatives, patients undergoing A-VATS experience a lower incidence of postoperative hypertension, tachycardia, and other perioperative complications than those under general anaesthesia, maintaining hemodynamic stability closer to baseline (35, 43). Spontaneous breathing in awake patients facilitates an immediate return to normal heart rate and blood pressure in the postoperative period, thereby reducing the risk of complications such as myocardial ischemia or arrhythmia. These findings support the effectiveness of A-VATS in improving perioperative outcomes and providing hemodynamic stability (35, 43). In our study, the change in heart rate from the preoperative to the intraoperative period was significantly greater in the A-VATS group than in the I-VATS group. Conversely, the postoperative systolic and diastolic blood pressures were significantly higher in the I-VATS group than in the A-VATS group. We believe that the observed intraoperative bradycardia and postoperative hypertension in the I-VATS group were due to the systemic effects of general anaesthesia and modifications inherent in A-VATS anaesthesia.

Postoperative body temperature was lower in the I-VATS group than in the A-VATS group. This difference is likely due to the loss of vascular tone and vasodilatory effects associated with general anaesthesia, while the drop in body temperature in both groups may be related to inadequate warming during or immediately after surgery. Previous studies have reported either significantly lower SpO₂ values in the A-VATS group compared to the I-VATS group (42) or no significant difference between the groups (33, 40, 44). Similarly, our study found no significant difference between the groups regarding preoperative/intraoperative SpO₂ changes. The fact that the intubated group was mechanically ventilated with a potentially higher oxygen fraction, while the awake group was managed under hypoxic-hypercapnic ventilation dynamics, suggests that the awake procedure does not pose an additional risk concerning oxygen saturation. This may be more helpful for patients with limited respiratory functions. The higher postoperative SpO₂ values observed in the A-VATS group relative to the preoperative levels may be explained by the fact that these patients continued to receive oxygen at a concentration similar to that administered during surgery.

Although minimally invasive surgical techniques have been shown to reduce operative complications compared to traditional methods (5), A-VATS, characterized by non-intubated techniques utilizing local anesthesia and sedation, has emerged as an appealing alternative to conventional intubated approaches, particularly for patients with compromised respiratory function or a higher risk of surgical stress (45). When comparing groups undergoing various procedures, some studies have reported similar complication rates between A-VATS and I-VATS (46, 47), whereas others have noted that the complication rate is significantly lower with A-VATS than with I-VATS (31). In another study evaluating pleural effusions, no significant difference was observed between the A-VATS and I-VATS groups regarding overall complications (23, 43). The avoidance of mechanical ventilation in A-VATS likely contributes to the reduced incidence of complications (48). Consistent with our findings, a retrospective study reported no significant difference in complications between the groups, except for a notable reduction in sore throat in the A-VATS group (24). In a meta-analysis comparing A-VATS and I-VATS complications, no difference was observed in the incidence of postoperative atelectasis in the A-VATS group (32). In contrast, we found that the incidence of atelectasis on postoperative day 0 was higher in the I-VATS group (*p* < 0.001). We speculate that this may be due to selective intubation and one-lung ventilation in the general anesthesia group, resulting in complete lung collapse. Even if surgical pneumothorax is induced in the awake group, airway patency is maintained, allowing for a more rapid adaptation to re-expansion. As routine positive-pressure ventilation before extubation was not administered in the intubated group, it remains unclear whether its use would have altered the incidence of atelectasis between the groups.

Several studies encompassing various procedures have reported that patients in the A-VATS group exhibit significantly higher anesthesia satisfaction scores compared to those in the I-VATS group (24, 29, 31, 35). In contrast, a prospective randomized controlled decortication study indicated significantly lower patient satisfaction in the A-VATS group than in the I-VATS group (42). In our study, anesthesia satisfaction was significantly higher with A-VATS. The elevated satisfaction scores associated with A-VATS may be attributed to the avoidance of the adverse effects of general anesthesia and intubation, as well as the benefits of early mobilization, prompt initiation of oral intake, rapid restoration of bowel function, and more effective pain control achieved with regional analgesia.

Studies have indicated that the duration of anesthesia was significantly shorter in the A-VATS group than in the I-VATS group (22, 29, 33, 40). A meta-analysis evaluating 970 patients who underwent lobectomy or segmentectomy reported shorter operative times in the A-VATS group (46). Conversely, a prospective randomized controlled study found no difference in the duration of surgery between A-VATS and I-VATS (29). Similarly, a meta-analysis by Zhang et al., which included 12 randomized controlled trials, noted that the mean operative time did not differ significantly between patients undergoing A-VATS and I-VATS (32). Our study also demonstrated no significant differences in the total anesthesia time or duration of surgery between the groups, which we attribute to the experience of the surgical teams, their coordinated collaboration, and meticulous preoperative planning.

Previous reports indicated that the length of hospital stay was significantly shorter in the A-VATS group than in the I-VATS group (23, 24, 32, 35, 46, 49). The reduced hospital stay associated with A-VATS is believed to stem from earlier initiation of oral intake and mobilization (50). In contrast, our study revealed that the hospital stay was significantly longer in the A-VATS group. Notably, among the nine patients with extended hospitalizations exceeding 10 days, two were in the intubated group and seven in the awake group, which were likely linked to issues such as persistent air leaks, additional interventions for infectious complications, and the presence of cardiovascular comorbidities.

In studies of A-VATS performed for pleural effusion, the duration of chest drainage was reported to be significantly shorter in the A-VATS group than in the intubated group (43, 51). However, a randomized controlled study on A-VATS decortication found no significant difference in the length of drain stay between groups (42). In our study, the length of drain stay was significantly longer in the A-VATS group, which may be attributed to the higher prevalence of previous pleural or parenchymal procedures and the more vulnerable clinical profile of the patients in this group.

We believe that the extended postoperative hospital stay noted in the A-VATS group is linked to the fact that patients are unsuitable for general anesthesia due to potential comorbidities; therefore, they underwent A-VATS. The prolonged duration of chest tube stay was a common cause in this group.

Many advantages, such as rapid recovery by avoiding general anesthesia, intubation, and mechanical ventilation, along with a quicker return to daily activities—such as early oral intake and mobilization—resulting from the absence of intubation-related discomfort like sore throat can be achieved with A-VATS (4, 35, 49, 52). The onset of oral intake (24, 44), ambulation (24), flatulence, or defecation (24) was significantly shorter in the A-VATS group than in the I-VATS group, and our findings are consistent with these studies. We believe that the earlier recovery observed in the awake group was due to the avoidance of general anesthesia procedures, such as intubation and neuromuscular blockade. Although all patients were managed according to the ERAS protocol postoperatively, quicker recovery in the awake group likely allowed for greater benefits from the ERAS protocol.

Previous studies have consistently reported that the overall cost of A-VATS is significantly lower than that of I-VATS, primarily due to reduced anesthesia requirements, shorter recovery periods, and fewer postoperative complications (22, 52). In our study, no statistically significant difference was observed in total hospitalization costs between the A-VATS and I-VATS groups. This finding is particularly noteworthy given that patients in the A-VATS cohort had significantly longer lengths of stay and required more intensive postoperative surveillance, largely due to a higher prevalence of comorbidities and elevated anesthetic risk profiles. These patients were specifically selected for A-VATS because they were deemed unsuitable candidates for general anesthesia and mechanical ventilation. Had these high-risk individuals undergone I-VATS, the probability of postoperative deterioration necessitating intensive care unit (ICU) admission and advanced supportive interventions would likely have increased, potentially resulting in substantially higher healthcare expenditures. The economic neutrality observed may be attributable to several compensatory factors associated with A-VATS, including reduced anesthesia-related costs, avoidance of intubation and mechanical ventilation, fewer ICU admissions, and decreased analgesic requirements, particularly with respect to opioid use. Thus, while the extended hospitalization duration in the A-VATS group might intuitively suggest increased costs, these were likely offset by the lower resource intensity of intraoperative and early postoperative care. Although the observed cost equivalence did not reach statistical significance, the application of A-VATS in high-risk surgical candidates appears to confer a protective effect against cost escalation by circumventing complications typically associated with general anesthesia in vulnerable populations. These findings underscore the potential clinical and economic value of A-VATS when used selectively in appropriately stratified patients. Future prospective cost-effectiveness analyses incorporating standardized perioperative protocols and broader patient populations are warranted to further substantiate these conclusions and guide evidence-based resource allocation in thoracic surgery.

It has been reported that minimizing surgical trauma helps preserve immune function (53, 54). Local anesthesia is less traumatic to the immune system and facilitates faster recovery (26). Surgical stress, extensive tissue trauma, and general anesthesia can reduce the circulating levels of lymphocytes and natural killer (NK) cells (55, 56). Surgical trauma, side effects of drugs used during general anesthesia, and impaired NK cell activity induced by one-lung ventilation (57, 58) contribute to immune dysfunction and increase susceptibility to postoperative infections (54, 56, 57). The reduced cytotoxic activity of peripheral lymphocytes may elevate the risk of tumor progression and metastasis (59), and alterations in postoperative wound healing may predispose patients to infections, thereby adversely affecting postoperative recovery (60, 61). The significantly lower postoperative inflammatory cell response in A-VATS patients compared to I-VATS patients may indicate better inflammatory regulation (62). Studies have shown that postoperative stress and inflammatory cell responses in A-VATS patients are relatively mild, likely due to the inhibition of the afferent and efferent sympathetic pathways (63). By mitigating stress and inflammatory responses, A-VATS may provide a safer surgical experience for high-risk patient populations (24).

In a prospective study, Vanni et al. A-VATS patients exhibited higher lymphocyte and NK cell counts 1 day post-surgery than I-VATS patients (63). A retrospective study indicated significantly lower postoperative white blood cell, neutrophil, and lymphocyte counts in the A-VATS group relative to the I-VATS group (24). In our study, the percentage changes in the pre-to postoperative absolute neutrophil (×10^3^) count, neutrophil (%), and percentage increase in neutrophil (%) were lower in the A-VATS group (*p* = 0.04, *p* = 0.04, and *p* = 0.02, respectively). The more pronounced neutrophil response observed in the I-VATS group suggests a more intense systemic inflammatory reaction in this cohort. The avoidance of general anesthesia and intubation, thus preventing exposure to a foreign body such as an endotracheal tube, even if sterile, and avoiding positive-pressure ventilation may have contributed to the lower neutrophil values observed in the A-VATS group (64, 65).

Parameters such as the Neutrophil-to-Lymphocyte Ratio (NLR) and Lymphocyte-to-Monocyte Ratio (LMR) have gained traction in recent years for assessing acute and chronic inflammation and adaptive immunity in cardiovascular diseases and various malignancies (66, 67). The Platelet-to-Lymphocyte Ratio (PLR), which reflects changes in platelet and lymphocyte counts due to acute inflammatory and prothrombotic conditions, is often used as a marker for neoplastic diseases and cardiovascular events (67). All three ratios have been used prognostically in various clinical scenarios involving inflammation. In our study, no statistically significant differences were found between the groups in the preoperative and postoperative changes in these ratios.

To the best of our knowledge, no study involving the same patient group has thoroughly evaluated all these parameters or examined the preoperative/postoperative changes in serum biochemical parameters within this context. The absence of significant differences in the percentage changes in these parameters between the groups indicated that the procedure did not lead to substantial alterations in the biochemical profiles.

This study has several inherent limitations that warrant consideration. First, the retrospective, single-center design and relatively small sample size may restrict the generalizability of the findings and limit the statistical power for detecting subtle differences between groups. A notable source of bias is the non-randomized allocation of patients, with A-VATS preferentially offered to individuals deemed unsuitable for general anesthesia due to compromised functional status or significant comorbidities. This selection bias may have influenced the observed perioperative outcomes, including the higher incidence of severe complications and prolonged hospital stays in the A-VATS group. Moreover, while patient cooperation was a prerequisite for inclusion in the A-VATS cohort, no standardized or objective assessment tool was employed to evaluate this criterion preoperatively. Only univariate analyses were conducted, which limits the ability to control for potential confounding factors. Although propensity score matching is a recognized method for mitigating bias in observational studies, its application was not feasible due to the limited sample size. Despite these methodological constraints, this study provides valuable insights by being, to our knowledge, the first to comprehensively assess a wide spectrum of clinical, physiological, and inflammatory parameters in a clearly defined patient population undergoing A-VATS vs. I-VATS. Future research should aim to validate these findings through larger, prospective, randomized controlled trials and consider multivariate or propensity-matched analyses to better elucidate the patient subgroups most likely to benefit from awake thoracic surgery techniques.

## 5 Conclusions

Based on our findings, A-VATS demonstrated several distinct advantages, including significantly lower postoperative pain scores, earlier achievement of recovery milestones such as oral intake, mobilization, and return of bowel function, and comparable hospitalization costs despite a longer length of hospital stay. These benefits were particularly notable in high-risk patients for whom conventional intubated approaches may pose greater perioperative risks. Furthermore, patient satisfaction scores and physiological outcomes in the A-VATS group remained favorable, even in the presence of a higher burden of comorbidities. However, the longer durations of hospitalization and chest tube placement, along with a higher incidence of severe complications, highlight the necessity for cautious patient selection and individualized perioperative planning. A-VATS holds considerable promise for broader adoption in routine thoracic surgical practice, particularly for selected patients with elevated anesthetic risk. To establish its optimal clinical role and ensure safe implementation, further prospective and randomized studies with larger sample sizes and standardized perioperative protocols are essential. These investigations will help refine patient selection criteria and support the development of evidence-based guidelines for the use of A-VATS in pleural surgery.

## Data Availability

The data analyzed in this study is subject to the following licenses/restrictions: the raw data supporting the conclusions of this article will be made available by the authors, without undue reservation. Requests to access these datasets should be directed to ezel.ersen@iuc.edu.tr.
